# Supplementary material for: Bioinformatic Approach to Identify Potential TGFB2-Dependent and Independent Prognostic Biomarkers for Ovarian Cancers Treated with Taxol
Source: Int J Mol Sci. 2025 Dec 10;26(24):11900. doi: 10.3390/ijms262411900 (PMC12733110; doi:10.3390/ijms262411900)
Supplement: Supplementary file 1 [file ijms-26-11900-s001.zip › ijms-3990680-supplementary.pdf]

Supplementary methods.

AI-Augmented Summaries of Pubmed Abstracts

Pubmed searches using the keywords: "Gene AND TCGA AND Prognosis AND Ovarian retrieved 787 abstracts; "Ovarian AND single-cell RNAseq" (767 abstracts); and "Ovarian AND TGF-beta" (1718 abstracts) (https://pubmed.ncbi.nlm.nih.gov/ accessed 17/12/24) were downloaded as text documents for processing using the Oncotelic Chatbot technologies. Each abstract was then indexed into our Quadrant database (aided using puppeteer 19.11.1), embedded, and transformed (langchain-openai 0.2.3, openai 1.52.0) into a vector of numbers capturing semantic similarity between text elements (tokens). The embedding transforms all abstracts to the same vector "embedding" space. It has been trained to minimize the distance (in vector space) between any pair of abstracts to the extent that they are semantically similar. In the question-answering session, the user query was transformed into an embedding vector. Then an appropriate similarity measure (eg. Cosine distance) was used to identify the embedded abstract vectors closest to the embedded query vector: the abstracts corresponding to these matching vectors were then fed to the question-answering model (in the form of context), along with the query, to arrive at an answer to the query.

The user interface is powered by the React framework, an open-source and flexible language for developing powerful front-end interfaces (https://react.dev/ accessed 25/03/2024). In addition, we used the @mui/material (https://mui.com/ accessed on 25/03/24)) libraries for the interface's design aspects and aimed to follow closely the material design guidelines. Serving the front end was Node.Js (https://nodejs.org/en Accessed 25/03/2024). Node.js libraries included in the project: @adobe/pdfservices-node-sdk 3.4.2@aws-sdk/client-s3 3.412.0, @langchain/community 0.2.5, @material-ui/core 4.12.4, @mui/base 5.0.0-beta.18, @mui/icons-material 5.11.16, @mui/material 5.15.20, @mui/styled-engine-sc 5.12.0, @mui/x-date-pickers 6.15.0, @qdrant/js-client-rest 1.4.0, carrot2 0.0.1, pdf2img 0.5.0, pdfjs-dist 4.5.136, puppeteer 19.11.1, react 18.2.0, sequelize 6.31.1, zod 3.22.4.

The AI-augmented interrogation of Pubmed enabled the rapid identification of key primary publications pertinent to this research project.

Identifying Prognostically Relevant Signaling Proteins Networked for TGFB2-dependent and TGFB2-independent Marker Genes Using the STRING Interaction Algorithm

Using the STRING Interaction Algorithm Putative protein–protein interaction (PPI) networks derived from mRNA levels with prognostic impact on the OS were constructed using the STRING version 12 algorithm (https://string-db.org/cgi/input?sessionId=bR8QWNAdLhxe&input_page_show_search=on, accessed 27th October 2025). In these diagrams, nodes represent protein identifiers while edges illustrate associations between proteins. Network visualization was achieved by seeding with a list of genes that showed significant upregulation in tumor tissue and demonstrated prognostic relevance for *TGFB2*-dependent and independent marker genes. The edges depicted the confidence level for each association, determined through multiple sources of experimental evidence, including text mining, laboratory experiments, databases, co-expression, neighborhood analysis, gene fusion, and co-occurrence (selected under "active interaction sources:" options on the web portal). Interaction edge scores exceeding 0.4 were used to define protein associations, with the thickness of connecting lines denoting score thresholds of 0.4, 0.7, and 0.9 (selected "medium confidence (0.400)" for "minimum required interaction score" option in the web interface). We only used the query proteins to generate the diagram (selected "none/query proteins only" under the "max number of interactors to show:" option in the web interface). Protein–protein interaction networks were clustered based on association scores using the Markov Clustering (MCL) algorithm in STRING (Inflation parameter = 1.3 set in the web interface); solid lines indicate connections within identified clusters, whereas dotted lines represent links between distinct clusters. Significance of the associations was assessed by calculating the number of nodes, the number of associations, the average node degree to derive the PPI enrichment p-value from the expected number of edges.

Flow chart for identification of prognostic markers

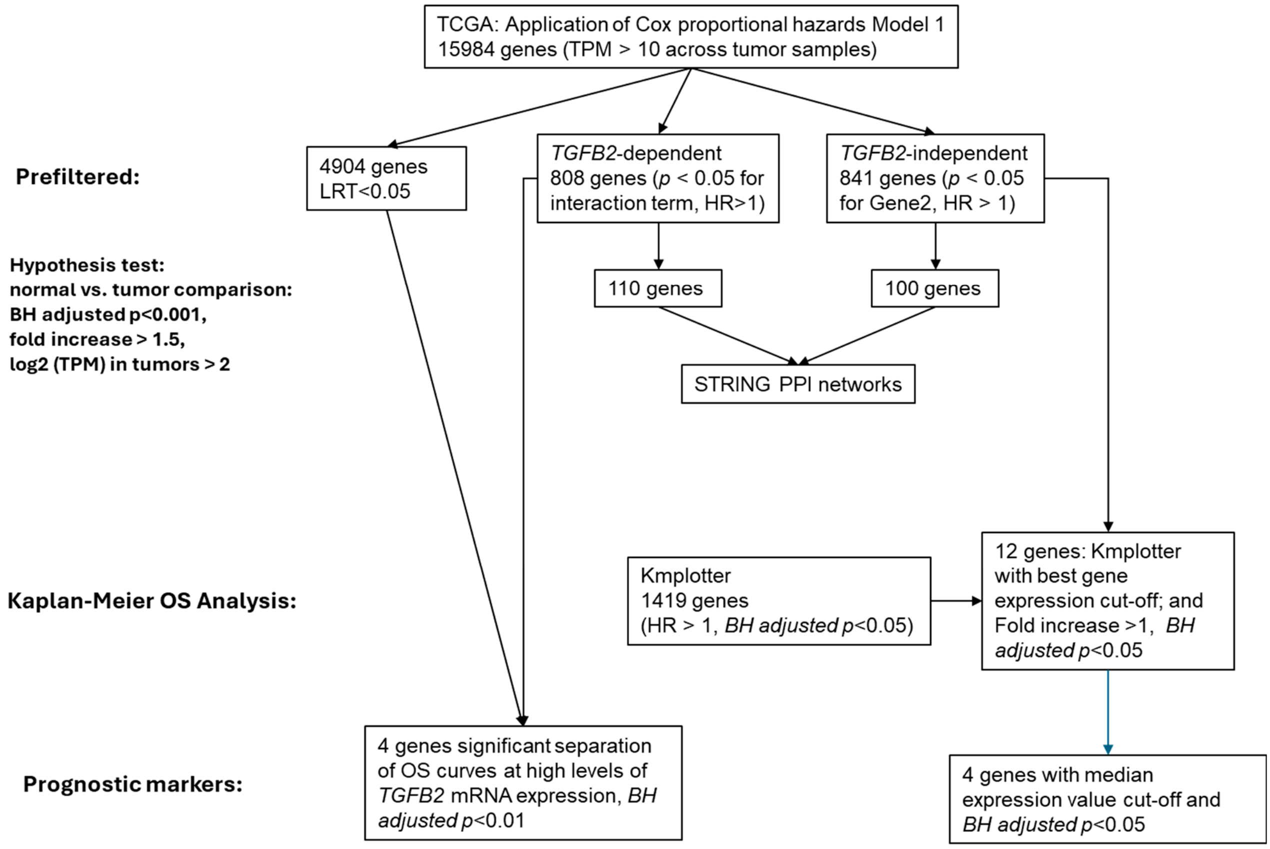

Table S1. *TGFB2*-dependent upregulated genes in tumors

Gene	Mean Log_2 TPM ± SEM		Fold Increase	Linear Contrast
	Normal	Tumor	Tumor/Normal	*Adj. p*-value
CLDN6	-8.28 ± 0.29	4.55 ± 0.21	7267.07	<0.0000001
KLK7	-3.6 ± 0.28	7.02 ± 0.13	1574.02	<0.0000001
MAL2	-2.12 ± 0.19	7.34 ± 0.1	705.00	<0.0000001
EPCAM	-0.57 ± 0.18	7.92 ± 0.1	359.89	<0.0000001
PRSS8	-1.72 ± 0.16	6.64 ± 0.11	327.72	<0.0000001
MYBL2	-3.32 ± 0.26	5.01 ± 0.1	321.06	<0.0000001
KRT7	0.15 ± 0.17	8.39 ± 0.11	303.36	<0.0000001
WNT10A	-3.95 ± 0.28	4.08 ± 0.14	261.12	<0.0000001
KRT8	3.01 ± 0.14	9.64 ± 0.1	98.78	<0.0000001
TJP3	-2.17 ± 0.26	4.11 ± 0.09	78.02	<0.0000001
POU2F3	-3.04 ± 0.23	3.19 ± 0.13	75.50	<0.0000001
PKMYT1	-1.34 ± 0.18	4 ± 0.08	40.33	<0.0000001
TMEM45B	-3.15 ± 0.16	2.11 ± 0.12	38.21	<0.0000001
ARHGAP8	-1.14 ± 0.23	4.07 ± 0.08	36.98	<0.0000001
KCNC3	-2.18 ± 0.17	2.92 ± 0.1	34.32	<0.0000001
UPK3B	1.23 ± 0.19	6.06 ± 0.16	28.45	<0.0000001
ERBB3	0.45 ± 0.12	5.17 ± 0.1	26.29	<0.0000001
PAQR4	-0.89 ± 0.16	3.62 ± 0.09	22.83	<0.0000001
TMEM184A	-1.55 ± 0.13	2.93 ± 0.1	22.31	<0.0000001
MMP15	-0.24 ± 0.13	4 ± 0.09	18.83	<0.0000001
LY6G6C	-1.71 ± 0.18	2.25 ± 0.16	15.57	<0.0000001
CRTAC1	-0.97 ± 0.14	2.86 ± 0.17	14.28	<0.0000001
SLC29A2	0.42 ± 0.09	4.18 ± 0.08	13.57	<0.0000001
CDCA7	-0.63 ± 0.11	3.11 ± 0.1	13.38	<0.0000001
KAZALD1	-1.25 ± 0.17	2.43 ± 0.13	12.87	<0.0000001
ANLN	-0.35 ± 0.1	3.23 ± 0.08	11.94	<0.0000001
CITED4	3.3 ± 0.05	6.3 ± 0.1	8.00	<0.0000001
ENTPD2	-0.26 ± 0.12	2.72 ± 0.11	7.87	<0.0000001
MAP7	0.32 ± 0.07	3.28 ± 0.08	7.77	<0.0000001
SPOCK2	2.48 ± 0.13	5.26 ± 0.13	6.86	<0.0000001
PTPN6	2.25 ± 0.09	5.01 ± 0.07	6.75	<0.0000001
PNP	2.27 ± 0.13	4.89 ± 0.09	6.13	<0.0000001
SNTB1	0.55 ± 0.1	3.09 ± 0.09	5.81	<0.0000001
IDH2	4.1 ± 0.06	6.64 ± 0.09	5.81	<0.0000001
CDCA3	2.25 ± 0.07	4.72 ± 0.09	5.54	<0.0000001
RYR1	-0.02 ± 0.11	2.44 ± 0.12	5.52	<0.0000001
ITGB8	1.91 ± 0.06	4.2 ± 0.1	4.89	<0.0000001
MTHFD2	3.83 ± 0.14	5.94 ± 0.09	4.32	<0.0000001
MMACHC	0.8 ± 0.06	2.74 ± 0.07	3.85	<0.0000001
SLC43A2	2.05 ± 0.09	3.96 ± 0.08	3.75	<0.0000001
ICAM1	3.05 ± 0.12	4.95 ± 0.11	3.74	<0.0000001
ZNF385A	2.48 ± 0.11	4.28 ± 0.08	3.47	<0.0000001
EPHA2	2.3 ± 0.1	4.08 ± 0.09	3.41	<0.0000001
RINL	0.76 ± 0.07	2.52 ± 0.09	3.40	<0.0000001
CYC1	6.17 ± 0.03	7.88 ± 0.09	3.27	<0.0000001
PLEKHH1	0.84 ± 0.09	2.55 ± 0.09	3.27	<0.0000001

ARF3	5.1 ± 0.04	6.76 ± 0.08	3.15	<0.0000001
ORAI1	3.01 ± 0.06	4.62 ± 0.07	3.06	<0.0000001
PPDPF	8.12 ± 0.05	9.71 ± 0.1	3.01	<0.0000001
PSMB2	4.89 ± 0.03	6.42 ± 0.08	2.89	<0.0000001
SNHG4	0.96 ± 0.11	2.42 ± 0.09	2.75	<0.0000001
NME1-NME2	2.4 ± 0.1	3.85 ± 0.09	2.73	<0.0000001
FJX1	2.46 ± 0.15	3.9 ± 0.07	2.70	<0.0000001
KIF9	2.35 ± 0.05	3.77 ± 0.08	2.69	<0.0000001
RAB11FIP1	1.54 ± 0.06	2.91 ± 0.08	2.59	<0.0000001
CD2AP	2.27 ± 0.04	3.64 ± 0.07	2.59	<0.0000001
ADA	1.68 ± 0.08	3.02 ± 0.07	2.52	<0.0000001
ESR1	2.83 ± 0.06	4.16 ± 0.11	2.52	<0.0000001
TRAF7	4.62 ± 0.05	5.93 ± 0.08	2.49	<0.0000001
GDF11	2.23 ± 0.07	3.53 ± 0.1	2.47	<0.0000001
LHPP	3.27 ± 0.06	4.56 ± 0.08	2.44	<0.0000001
ZNF101	1.19 ± 0.05	2.44 ± 0.07	2.39	<0.0000001
ABCC4	1.89 ± 0.06	3.15 ± 0.09	2.39	<0.0000001
SDHB	5.45 ± 0.04	6.69 ± 0.08	2.36	<0.0000001
TRAIP	1.59 ± 0.06	2.81 ± 0.07	2.32	<0.0000001
FAHD1	2.68 ± 0.05	3.88 ± 0.07	2.30	<0.0000001
DSN1	2.26 ± 0.05	3.46 ± 0.07	2.29	<0.0000001
MBOAT7	5.24 ± 0.05	6.43 ± 0.08	2.29	<0.0000001
MAP2K3	3.76 ± 0.06	4.9 ± 0.07	2.21	<0.0000001
TRPM4	2.48 ± 0.1	3.6 ± 0.09	2.17	<0.0000001
TMEM123	5.57 ± 0.06	6.66 ± 0.09	2.13	<0.0000001
XRCC4	1.11 ± 0.06	2.19 ± 0.07	2.12	<0.0000001
CPNE2	4.21 ± 0.05	5.29 ± 0.09	2.11	<0.0000001
TNNC1	1.01 ± 0.14	2.07 ± 0.16	2.09	0.0000001
CLN6	3.64 ± 0.04	4.65 ± 0.07	2.01	0.0000003
MSX1	1.25 ± 0.09	2.23 ± 0.15	1.97	0.0000005
NDUFA1	6.26 ± 0.02	7.23 ± 0.08	1.95	0.0000008
TRAPPC5	5.56 ± 0.03	6.53 ± 0.08	1.95	0.0000009
BTF3L4	5.17 ± 0.03	6.13 ± 0.08	1.94	0.000001
RAB32	3.51 ± 0.06	4.44 ± 0.08	1.90	0.0000021
LIMK1	3.77 ± 0.05	4.7 ± 0.08	1.90	0.0000024
PIP5K1A	4.77 ± 0.04	5.68 ± 0.08	1.87	0.0000036
NDUFS3	5.69 ± 0.04	6.6 ± 0.08	1.87	0.0000037
SEC23B	3.88 ± 0.06	4.78 ± 0.07	1.86	0.0000049
GSPT1	4.92 ± 0.03	5.8 ± 0.08	1.84	0.0000066
PARP8	2.75 ± 0.04	3.63 ± 0.08	1.84	0.0000076
TMEM87B	2.45 ± 0.05	3.32 ± 0.07	1.83	0.0000086
FGFR2	3.64 ± 0.12	4.51 ± 0.09	1.83	0.0000088
PHACTR4	3.76 ± 0.04	4.62 ± 0.08	1.81	0.0000127
CAMTA1	5.71 ± 0.03	6.55 ± 0.08	1.79	0.0000171
RAB15	1.48 ± 0.1	2.31 ± 0.08	1.78	0.0000214
APEX1	6.91 ± 0.03	7.74 ± 0.08	1.78	0.0000219
TIGD5	1.91 ± 0.07	2.73 ± 0.07	1.76	0.000029
SLC6A8	3.62 ± 0.11	4.44 ± 0.11	1.76	0.000031
MOSPD3	4.61 ± 0.03	5.41 ± 0.07	1.74	0.0000456
DR1	3.48 ± 0.05	4.27 ± 0.07	1.72	0.0000595
UCHL5	3.38 ± 0.04	4.16 ± 0.07	1.71	0.0000741
RAB18	4.61 ± 0.04	5.38 ± 0.07	1.71	0.0000814
ANP32A	5.78 ± 0.04	6.54 ± 0.08	1.70	0.0000923
FAM174B	3.29 ± 0.06	4.03 ± 0.08	1.67	0.0001583

CAPNS1	8.58 ± 0.03	9.32 ± 0.09	1.67	0.0001675
STAU2	3.7 ± 0.03	4.43 ± 0.07	1.66	0.0001787
MAZ	6.74 ± 0.04	7.45 ± 0.08	1.64	0.0002645
PHC3	3.08 ± 0.04	3.78 ± 0.08	1.63	0.0002978
CCNY	4.56 ± 0.04	5.26 ± 0.08	1.62	0.0003854
NDUFS1	3.91 ± 0.05	4.59 ± 0.07	1.60	0.0005064
BORCS8	3.82 ± 0.04	4.49 ± 0.07	1.59	0.0006344
YTHDF2	4.71 ± 0.04	5.36 ± 0.07	1.57	0.0008556
SPNS1	4.64 ± 0.03	5.29 ± 0.07	1.57	0.0008699
SLC9A3-OT1	3.76 ± 0.04	4.41 ± 0.08	1.56	0.0009772

Table S2. Significant statistical interaction determined from Cox proportional hazards model for *TGFB2* and Marker Gene mRNA upregulated in tumor tissues

Gene 2	*TGFB2* mRNA (Zscore)		Gene 2 mRNA (Z score)		Age (yrs)		Chemo vs Others		TGFB2 by Gene 2 Interaction	
	HR(95% CI)	*p*–val	HR(95% CI)	*p*–val	HR(95% CI)	*p*–val	HR(95% CI)	*p*–val	HR(95% CI)	*p*–val
HOXC4	1.06 (0.85-1.3)	0.618	1.36 (1.03-1.78)	0.028	1.02 (1-1.03)	0.058	1.41 (0.93-2.14)	0.107	1.74 (1.19-2.56)	0.004
MAL2	0.9 (0.72-1.12)	0.335	1 (0.82-1.23)	0.974	1.02 (1-1.04)	0.024	1.28 (0.84-1.96)	0.249	0.79 (0.62-1)	0.046
STAU2	0.94 (0.78-1.14)	0.544	0.99 (0.87-1.13)	0.925	1.02 (1-1.03)	0.045	1.44 (0.95-2.2)	0.085	1.34 (1.07-1.67)	0.011
TRPV4	1.09 (0.88-1.35)	0.44	1.26 (1.03-1.55)	0.027	1.02 (1-1.03)	0.054	1.34 (0.88-2.03)	0.174	1.53 (1.1-2.14)	0.012

Table S3. Kaplan Meier analysis comparing median OS (months) of *TGFB2*/Marker Gene with 50 percentile cut-offs for patient subgroupings.

**Gene 2**	**Group1**	***n***	**median OS**	**Group2**	***n***	**median OS**	***Adj. p*-val**
HOXC4	*TGFB2^{high}/Gene2^{low}*	57	57.4	*TGFB2^{high}/Gene2^{high}*	64	38.2	0.0091
HOXC4	*TGFB2^{high}/Gene2^{low}*	57	57.4	*TGFB2^{low}/Gene2^{low}*	63	44.8	0.0773
HOXC4	*TGFB2^{high}/Gene2^{low}*	57	57.4	*TGFB2^{low}/Gene2^{high}*	57	48.8	0.5796
HOXC4	*TGFB2^{high}/Gene2^{high}*	64	38.2	*TGFB2^{low}/Gene2^{low}*	63	44.8	0.3302
HOXC4	*TGFB2^{high}/Gene2^{high}*	64	38.2	*TGFB2^{low}/Gene2^{high}*	57	48.8	0.0676
HOXC4	*TGFB2^{low}/Gene2^{low}*	63	44.8	*TGFB2^{low}/Gene2^{high}*	57	48.8	0.3302
MAL2	*TGFB2^{high}/Gene2^{low}*	65	35.9	*TGFB2^{high}/Gene2^{high}*	56	59.1	0.0046
MAL2	*TGFB2^{high}/Gene2^{low}*	65	35.9	*TGFB2^{low}/Gene2^{low}*	55	44.9	0.1407
MAL2	*TGFB2^{high}/Gene2^{low}*	65	35.9	*TGFB2^{low}/Gene2^{high}*	65	48.3	0.1135
MAL2	*TGFB2^{high}/Gene2^{high}*	56	59.1	*TGFB2^{low}/Gene2^{low}*	55	44.9	0.1407
MAL2	*TGFB2^{high}/Gene2^{high}*	56	59.1	*TGFB2^{low}/Gene2^{high}*	65	48.3	0.1407
MAL2	*TGFB2^{low}/Gene2^{low}*	55	44.9	*TGFB2^{low}/Gene2^{high}*	65	48.3	0.9694
STAU2	*TGFB2^{high}/Gene2^{low}*	63	57.1	*TGFB2^{high}/Gene2^{high}*	58	35.0	0.0068
STAU2	*TGFB2^{high}/Gene2^{low}*	63	57.1	*TGFB2^{low}/Gene2^{low}*	57	47.6	0.1267
STAU2	*TGFB2^{high}/Gene2^{low}*	63	57.1	*TGFB2^{low}/Gene2^{high}*	63	47.5	0.4020
STAU2	*TGFB2^{high}/Gene2^{high}*	58	35.0	*TGFB2^{low}/Gene2^{low}*	57	47.6	0.3322
STAU2	*TGFB2^{high}/Gene2^{high}*	58	35.0	*TGFB2^{low}/Gene2^{high}*	63	47.5	0.0635
STAU2	*TGFB2^{low}/Gene2^{low}*	57	47.6	*TGFB2^{low}/Gene2^{high}*	63	47.5	0.5491
TRPV4	*TGFB2^{high}/Gene2^{low}*	63	58.2	*TGFB2^{high}/Gene2^{high}*	58	38.0	0.0011
TRPV4	*TGFB2^{high}/Gene2^{low}*	63	58.2	*TGFB2^{low}/Gene2^{low}*	57	43.7	0.1064
TRPV4	*TGFB2^{high}/Gene2^{low}*	63	58.2	*TGFB2^{low}/Gene2^{high}*	63	47.6	0.1600
TRPV4	*TGFB2^{high}/Gene2^{high}*	58	38.0	*TGFB2^{low}/Gene2^{low}*	57	43.7	0.0626
TRPV4	*TGFB2^{high}/Gene2^{high}*	58	38.0	*TGFB2^{low}/Gene2^{high}*	63	47.6	0.0564
TRPV4	*TGFB2^{low}/Gene2^{low}*	57	43.7	*TGFB2^{low}/Gene2^{high}*	63	47.6	0.6817

Table S4. *TGFB2*-independent upregulated genes in tumors

Gene	Mean Log_2 TPM ± SEM Normal	Tumor	Fold Increase Tumor/Normal	Linear Contrast *Adj. p*-value
FOXQ1	-7.22± 0.33	3.09± 0.14	1272.84	<0.0000001
MMP7	-4.42± 0.4	6.22± 0.17	1598.28	<0.0000001
PRSS21	-3.3± 0.34	4.54± 0.17	228.02	<0.0000001
KLHL14	-1.88± 0.11	4.06± 0.1	61.63	<0.0000001
CRB2	-3.58± 0.23	2.2± 0.13	55.04	<0.0000001
SLC4A11	-1.76± 0.13	3.46± 0.12	37.21	<0.0000001
SCNN1A	2.1± 0.12	7.29± 0.1	36.62	<0.0000001
TMC4	0.99± 0.08	5.97± 0.09	31.57	<0.0000001
LSR	2.7± 0.07	7.57± 0.09	29.19	<0.0000001
ARL4C	0.55± 0.14	5.27± 0.1	26.37	<0.0000001
GGT6	-1.16± 0.14	3.41± 0.11	23.78	<0.0000001
ADORA1	-2.38± 0.17	2.03± 0.09	21.32	<0.0000001
CLIC3	-0.75± 0.2	3.51± 0.12	19.25	<0.0000001
ANO9	-0.54± 0.15	3.66± 0.1	18.35	<0.0000001
HPN	0.3± 0.12	4.12± 0.17	14.20	<0.0000001
MISP3	-0.36± 0.11	3.35± 0.1	13.05	<0.0000001
NINJ2	-0.51± 0.14	3.06± 0.1	11.89	<0.0000001
CD9	5.62± 0.11	9.15± 0.1	11.54	<0.0000001
ANPEP	0.19± 0.18	3.54± 0.13	10.16	<0.0000001
NTN1	-0.89± 0.13	2.4± 0.11	9.78	<0.0000001
KIF1A	-0.07± 0.14	3.14± 0.18	9.25	<0.0000001
STRA6	-0.13± 0.15	3.03± 0.12	8.95	<0.0000001
MXRA5	1.04± 0.14	4.08± 0.12	8.20	<0.0000001
TMEM119	-0.82± 0.17	2.03± 0.12	7.19	<0.0000001
RASSF9	-0.37± 0.13	2.46± 0.08	7.08	<0.0000001
AHNAK2	0.83± 0.16	3.64± 0.11	7.03	<0.0000001
VCAN	0.98± 0.18	3.72± 0.14	6.71	<0.0000001
SLC6A12	-0.36± 0.13	2.31± 0.11	6.36	<0.0000001
GINS4	-0.35± 0.07	2.31± 0.08	6.31	<0.0000001
SPINT2	6.52± 0.06	9.08± 0.1	5.90	<0.0000001
SLC7A1	1.3± 0.1	3.85± 0.08	5.85	<0.0000001
CILP2	-0.27± 0.18	2.27± 0.11	5.83	<0.0000001
IL27RA	-0.16± 0.11	2.26± 0.1	5.34	<0.0000001
ZSWIM4	1.74± 0.05	4.11± 0.09	5.15	<0.0000001
RCOR2	0.17± 0.11	2.39± 0.12	4.66	<0.0000001
GAPDH	10.11± 0.04	12.21± 0.1	4.29	<0.0000001
TGM1	1.32± 0.11	3.41± 0.12	4.27	<0.0000001
TMEM33	2.53± 0.05	4.4± 0.07	3.66	<0.0000001
UNC5B	1.65± 0.09	3.52± 0.09	3.64	<0.0000001
FXYD5	4.56± 0.1	6.36± 0.09	3.49	<0.0000001
PODNL1	1.39± 0.13	3.18± 0.12	3.47	<0.0000001
DERA	2.9± 0.05	4.69± 0.08	3.46	<0.0000001
TIAM1	0.59± 0.13	2.3± 0.09	3.29	<0.0000001
PPL	2.5± 0.13	4.21± 0.1	3.26	<0.0000001
TGFBI	4.33± 0.12	5.95± 0.1	3.08	<0.0000001
AGAP1	1.95± 0.03	3.52± 0.08	2.97	<0.0000001
ORAI2	2.18± 0.05	3.74± 0.08	2.94	<0.0000001
TPI1	8.12± 0.06	9.57± 0.09	2.73	<0.0000001
TMEM38A	1.34± 0.06	2.75± 0.09	2.66	<0.0000001

VSIG4	2.79± 0.22	4.13± 0.11	2.53	<0.0000001
KIAA1217	3.4± 0.07	4.73± 0.08	2.52	<0.0000001
DUSP2	1.99± 0.16	3.29± 0.09	2.47	<0.0000001
SLC35E1	3.67± 0.03	4.96± 0.08	2.44	<0.0000001
CMTM4	2.27± 0.03	3.54± 0.08	2.42	<0.0000001
EMP1	3.99± 0.15	5.25± 0.1	2.39	<0.0000001
MANSC1	3.47± 0.06	4.68± 0.08	2.31	<0.0000001
EPHB2	2.73± 0.1	3.93± 0.1	2.29	<0.0000001
NCS1	2.94± 0.09	4.14± 0.08	2.29	<0.0000001
MS4A7	1.61± 0.17	2.8± 0.09	2.28	<0.0000001
PLXDC2	2.95± 0.08	4.14± 0.08	2.28	<0.0000001
CNNM4	2.3± 0.03	3.45± 0.07	2.21	<0.0000001
MAP1S	4.37± 0.05	5.5± 0.08	2.19	<0.0000001
DFFA	3.38± 0.03	4.46± 0.07	2.11	<0.0000001
SLC7A5	2.68± 0.12	3.72± 0.09	2.06	<0.0000001
IQCA1	1.5± 0.11	2.52± 0.1	2.04	0.0000000
OPA3	1.45± 0.07	2.45± 0.07	1.99	0.0000001
STAU1	4.98± 0.03	5.97± 0.08	1.99	0.0000001
ANGPTL4	1.68± 0.22	2.65± 0.11	1.97	0.0000001
SLC2A9	1.27± 0.06	2.25± 0.08	1.97	0.0000001
ELF4	3.55± 0.05	4.52± 0.08	1.97	0.0000001
VASP	4.62± 0.07	5.58± 0.08	1.95	0.0000001
CIB2	2.64± 0.06	3.6± 0.09	1.95	0.0000002
CSNK1G1	1.46± 0.04	2.41± 0.07	1.94	0.0000002
FCGBP	1.21± 0.21	2.12± 0.13	1.88	0.0000007
PUS7	2.32± 0.06	3.19± 0.07	1.83	0.0000020
LPCAT3	4.36± 0.07	5.2± 0.08	1.79	0.0000044
ADO	2.49± 0.06	3.32± 0.07	1.78	0.0000055
RPRD2	4.05± 0.04	4.87± 0.08	1.77	0.0000067
WBP11	4.54± 0.04	5.36± 0.08	1.77	0.0000075
TPM4	8.2± 0.06	9.02± 0.09	1.77	0.0000076
CRTC1	3.18± 0.04	3.99± 0.08	1.76	0.0000087
PORCN	3.25± 0.04	4.07± 0.1	1.76	0.0000092
SH3BP2	4.1± 0.05	4.9± 0.08	1.74	0.0000140
RARG	4.56± 0.06	5.35± 0.08	1.73	0.0000152
RIN2	3.56± 0.04	4.33± 0.08	1.71	0.0000242
TMEM127	4.68± 0.05	5.45± 0.08	1.70	0.0000270
NDE1	3.09± 0.05	3.84± 0.08	1.68	0.0000441
C5AR1	1.93± 0.13	2.68± 0.09	1.68	0.0000477
FASTKD5	2.53± 0.05	3.27± 0.07	1.66	0.0000616
MLF2	7.15± 0.03	7.85± 0.08	1.62	0.0001393
SLC9A1	3.47± 0.04	4.16± 0.08	1.61	0.0001601
RPL28	11.3± 0.04	11.99± 0.1	1.61	0.0001635
AP3M1	3.66± 0.04	4.33± 0.07	1.59	0.0002372
MED26	2.58± 0.04	3.24± 0.07	1.59	0.0002790
NFIX	5.54± 0.05	6.19± 0.09	1.57	0.0003675
COL8A2	1.58± 0.08	2.22± 0.11	1.55	0.0005254
BCL2L11	3.21± 0.06	3.85± 0.07	1.55	0.0005496
RPL18A	11.76± 0.03	12.37± 0.11	1.53	0.0008156
CBLL1	3.36± 0.05	3.97± 0.07	1.53	0.0008814
GGCX	3.83± 0.04	4.43± 0.07	1.52	0.0009445

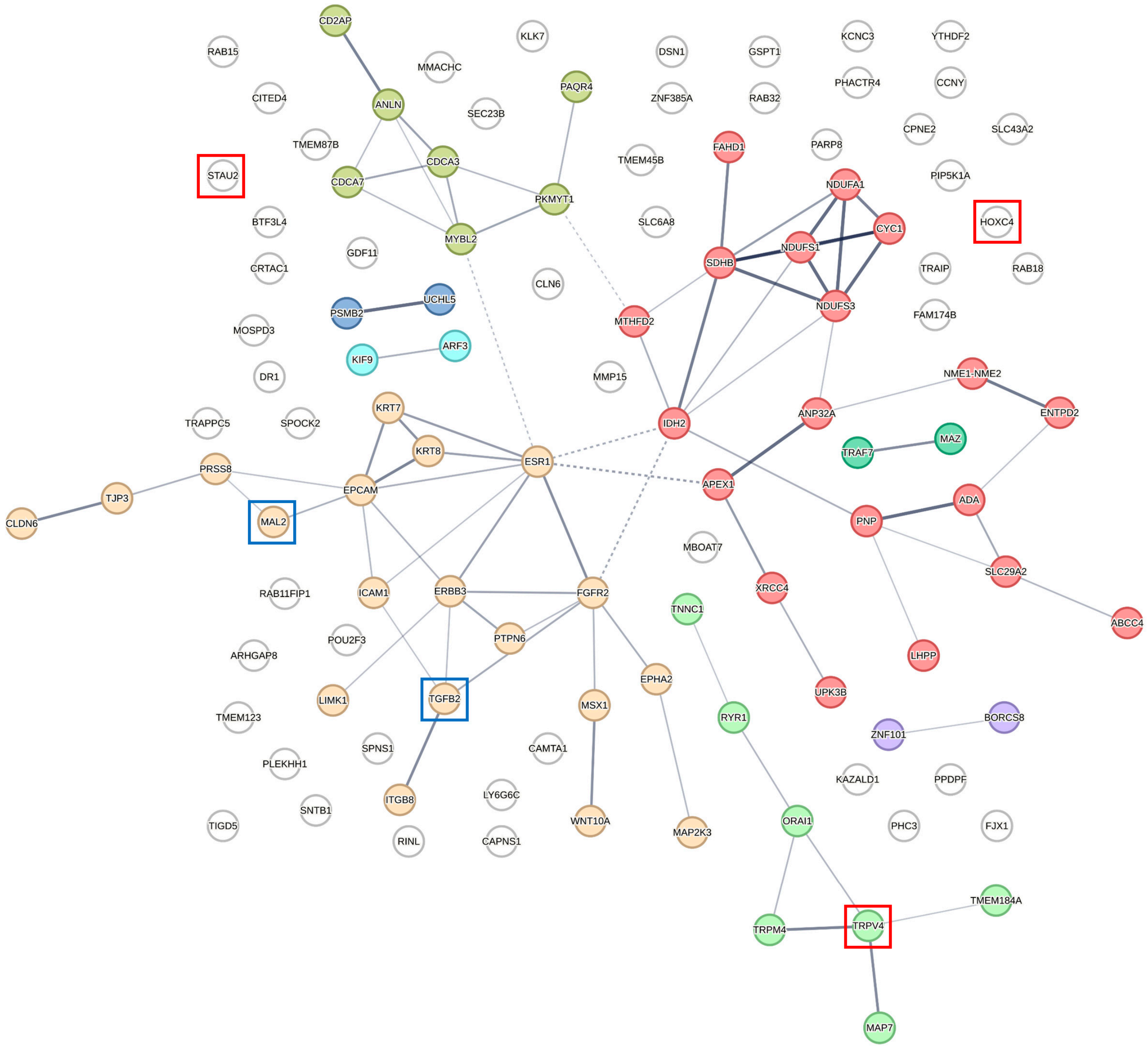

Figure S1. Protein-Protein Interaction networks for Prognostic markers for Ovarian cancer dependent on *TGFB2* mRNA expression. Depicted is a model STRING network comprised of marker gene mRNA nodes with prognostically significant impacts on OS associated with their high-level *TGFB2* mRNA expression. The network graph illustrates 111 protein nodes represented by 110 TGFB2 mRNA-interacting marker genes plus *TGFB2* mRNA with 83 edges, which were significantly higher than the expected number of 53 edges ($p < 0.0001$) based on a random set of proteins of the same size and degree of distribution drawn from the genome, suggesting that this highly connected set of nodes is biologically meaningful (solid lines depict the within-cluster associations and dotted lines depict between-cluster associations). The MCL clustering algorithm identified 8 clusters with enriched associations between nodes (solid edges): 4 clusters with two-gene associations, 2 clusters of 7 nodes each, and 2 clusters with 19 nodes each. TGFB2 appeared in the 19-node cluster with MAL2 (light brown nodes highlighted by blue squares). Ovarian cancer patients expressing high levels of *TGFB2* and *MAL2* mRNA exhibited the most improved OS curves. This contrasted with three genes that showed the greatest improvement in OS at high levels of *TGFB2*, in combination with low levels of *TRPV4*, *STAU2*, and *HOXC4* marker genes (highlighted by red squares). These 3 marker genes did not exhibit associations with the 2 main 19-node clusters. TRPV4 formed a cluster that included 7 proteins (green nodes), whereas STAU2 and HOXC4 formed no associations with the 8 clusters.

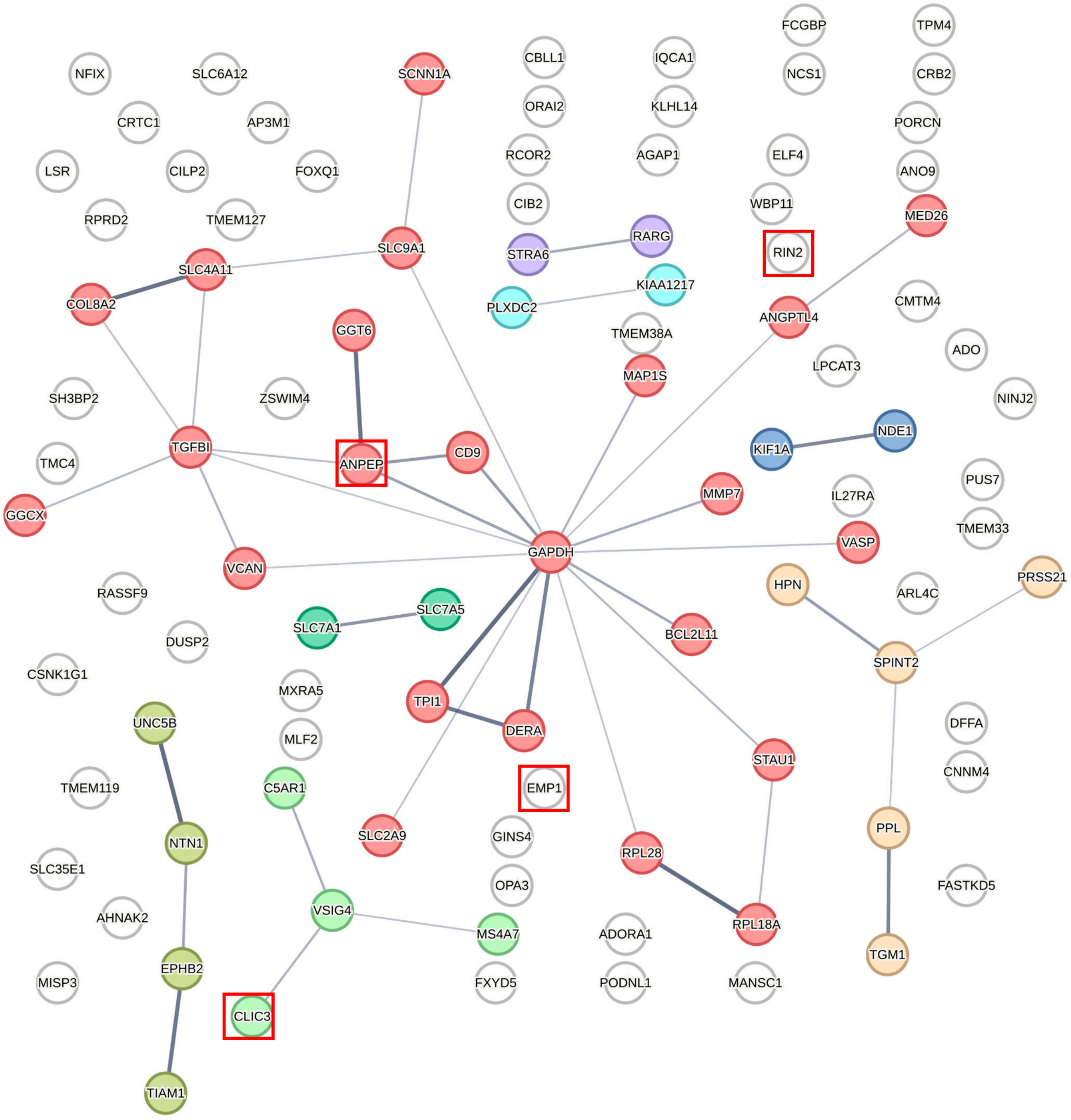

Figure S2. Protein-Protein Interaction Networks for Prognostic Markers for Ovarian Cancer, independent of *TGFB2* mRNA Expression. Depicted is a model STRING network comprised of marker gene mRNA nodes with prognostically significant impacts on OS associated with independent *TGFB2* mRNA expression. The network graph illustrates 100 nodes with 43 edges, which were significantly higher than the expected 30 edges ($p = 0.0186$) based on a random set of proteins of the same size and distribution in the genome, suggesting that this is a highly connected set of biologically meaningful nodes. (Solid lines depict the within-cluster associations and dotted lines depict between-cluster associations.) The MCL clustering algorithm identified 8 clusters with enriched associations between nodes (solid edges): 4 clusters with two-gene associations, 2 clusters of 4 nodes each, and a cluster of 5 nodes. ANPEP appeared in the 23-node cluster (red nodes highlighted by red squares). CLIC3 appeared in a 4-node cluster that was not connected to the main 23-node cluster. EMP1 and RIN2 showed no associations with any of the 8 clusters.

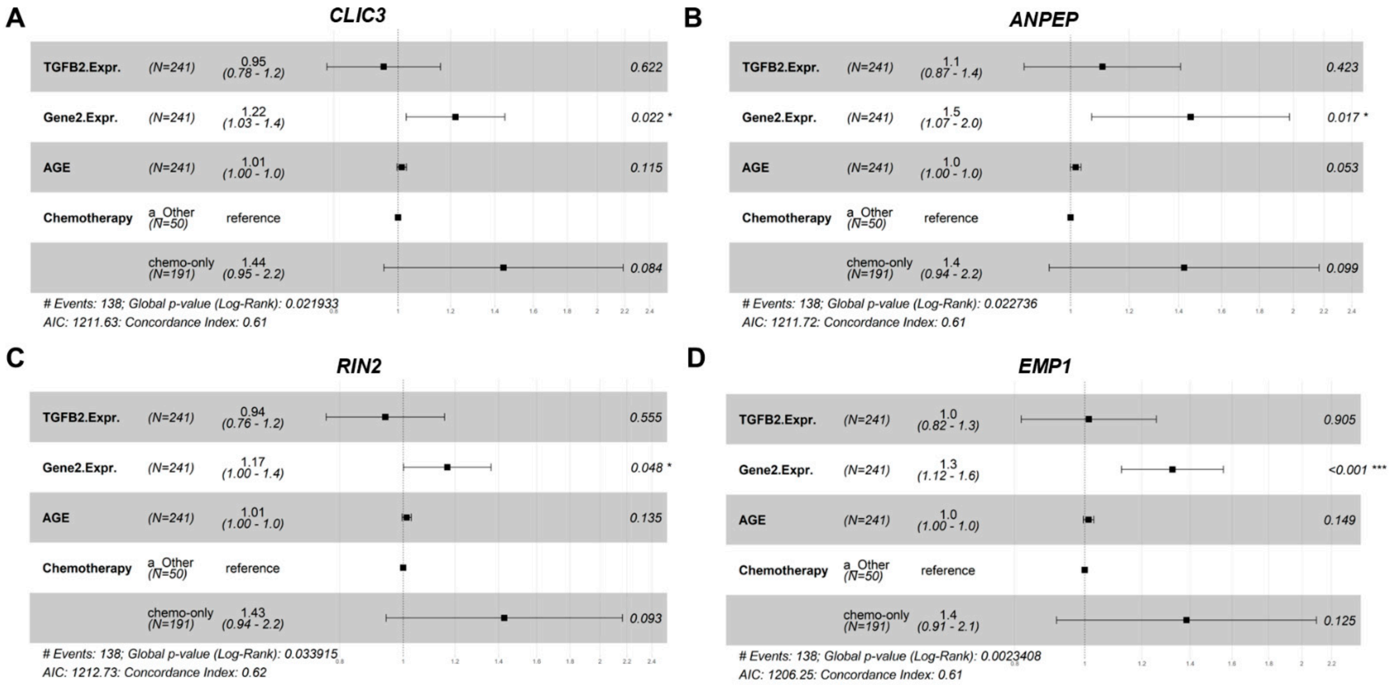

Figure S3. The impact of chemo-only treatment on *TGFB2* mRNA-independent prognostic markers identified from the multivariate Cox proportional hazards model in the TCGA dataset cross referenced with KMplotter dataset. Multivariate analyses used the Cox proportional hazards model to assess the individual effects of *TGFB2*, Gene2 marker mRNA expression levels (A. *CLIC3*; B. *ANPEP*; C. *RIN2*; D. *EMP1*), and the *TGFB2*-dependent OS impact via the interaction term in the multivariate model (Model 1). This analysis was controlled for age at diagnosis (Mean age = 59 yr) and treatment (chemo-only versus all other treatments). Model 1 included (i) *TGFB2* mRNA levels as a linear covariate expressed as Zscores (n = 241), (ii) The mRNA expression level for Gene2 mRNA levels as a linear covariate expressed as Zscores (n = 241), (iii) *TGFB2* by Gene2 interaction term to determine the dependency of Gene2 prognostic impact on *TGFB2* mRNA levels (n = 241), (iv). Treatment (Chemotherapy) comparing chemo-only (n = 191) versus other (n = 50) treatment regimens, and (v). Age expressed as a linear covariate (n = 241). The forest plots depict the HR calculations (95% CI) for each of the parameters of the model (* indicates $p < 0.05$, *** indicated $p < 0.001$). Model fits were reported by the AIC and LRT p-values ("Global p-value (Log-Rank)"). [A] There was a significant increase in HR as *CLIC3* expression levels increased (HR (95% CI range) = 1.22 (1.03-1.45); p = 0.022). There was not a significant impact on HR for the *TGFB2* by *CLIC3* interaction parameter (HR (95% CI range) = 0.95 (0.74-1.23); p = 0.715). [B] There was a significant increase in HR as *ANPEP* expression levels increased (HR (95% CI range) = 1.45 (1.07-1.98); p = 0.017). There was no significant impact on HR for the *TGFB2* by *ANPEP* interaction parameter (HR (95% CI range) = 1.79 (0.96-3.32); p = 0.066). [C] There was a borderline significant increase in HR as *RIN2* expression levels increased (HR (95% CI range) = 1.17 (1-1.36); p = 0.048). There was not a significant impact on HR for the *TGFB2* by *RIN2* interaction parameter (HR (95% CI range) = 1.02 (0.84-1.24); p = 0.869). [D] There was a significant increase in HR as *EMP1* expression levels increased (HR (95% CI range) = 1.32 (1.12-1.56); $p < 0.001$). There was not a significant impact on HR for the *TGFB2* by *EMP1* interaction parameter (HR (95% CI range) = 1.26 (0.93-1.7); p = 0.136).

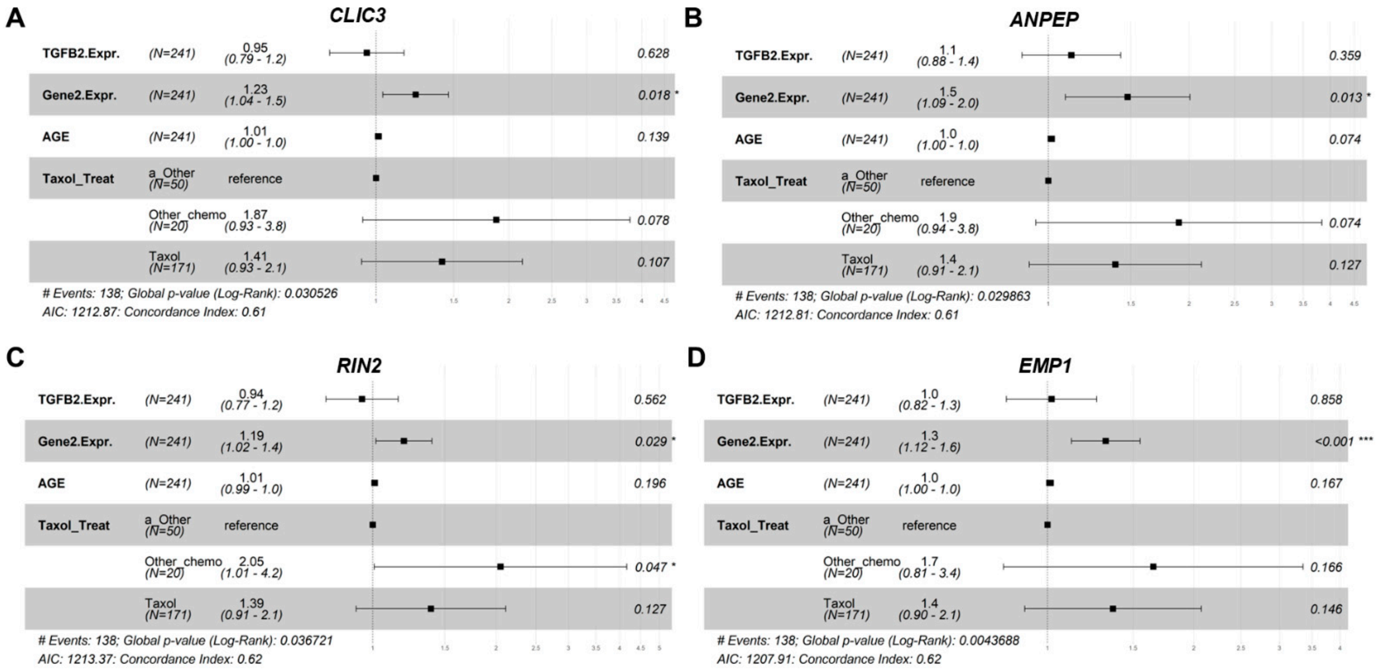

Figure S4. The impact of patients exposed to Taxol-containing treatments on *TGFB2* mRNA-independent prognostic markers identified from the multivariate Cox proportional hazards model in the TCGA dataset, cross-referenced with the KMplotter dataset. Multivariate analyses used the Cox proportional hazards model to assess the individual effects of *TGFB2*, Gene2 marker mRNA expression levels (A. *CLIC3*; B. *ANPEP*; C. *RIN2*; D. *EMP1*), and the *TGFB2*-dependent OS impact via the interaction term in the multivariate model (Model 2). This analysis was controlled for age at diagnosis (Mean age = 59 yr) and treatment (Taxol, other chemo and other treatments). Model 2 included (i) *TGFB2* mRNA levels as a linear covariate expressed as Zscores (n = 241), (ii) The mRNA expression level for Gene2 mRNA levels as a linear covariate expressed as Zscores (n = 241), (iii) *TGFB2* by Gene2 interaction term to determine the dependency of Gene2 prognostic impact on *TGFB2* mRNA levels (n = 241), (iv). Treatment (Taxol_Treat) containing Taxol (n = 171), other chemotherapies (Other_chemo) (n = 20) versus other (n = 50) treatment regimens, and (v). Age expressed as a linear covariate (n = 241). The forest plots depict the HR calculations (95% CI) for each of the parameters of the model (* indicates $p < 0.05$, *** indicated $p < 0.001$). Model fits were reported by the AIC and LRT p-values ("Global p-value (Log-Rank)"). [A] There was a significant increase in HR as *CLIC3* expression levels increased (HR (95% CI range) = 1.23 (1.04-1.46); $p = 0.018$). There was not a significant impact on HR for the *TGFB2* by *CLIC3* interaction parameter (HR (95% CI range) = 0.96 (0.75-1.23); $p = 0.76$). [B] There was a significant increase in HR as *ANPEP* expression levels increased (HR (95% CI range) = 1.48 (1.09-2.01); $p = 0.013$). There was not a significant impact on HR for the *TGFB2* by *ANPEP* interaction parameter (HR (95% CI range) = 1.84 (1-3.41); $p = 0.052$). [C] There was a significant increase in HR as *RIN2* expression levels increased (HR (95% CI range) = 1.19 (1.02-1.39); $p = 0.029$). There was not a significant impact on HR for the *TGFB2* by *RIN2* interaction parameter (HR (95% CI range) = 1.02 (0.84-1.23); $p = 0.846$). [D] There was a significant increase in HR as *EMP1* expression levels increased (HR (95% CI range) = 1.32 (1.12-1.55); $p < 0.001$). There was not a significant impact on HR for the *TGFB2* by *EMP1* interaction parameter (HR (95% CI range) = 1.28 (0.94-1.73); $p = 0.114$).

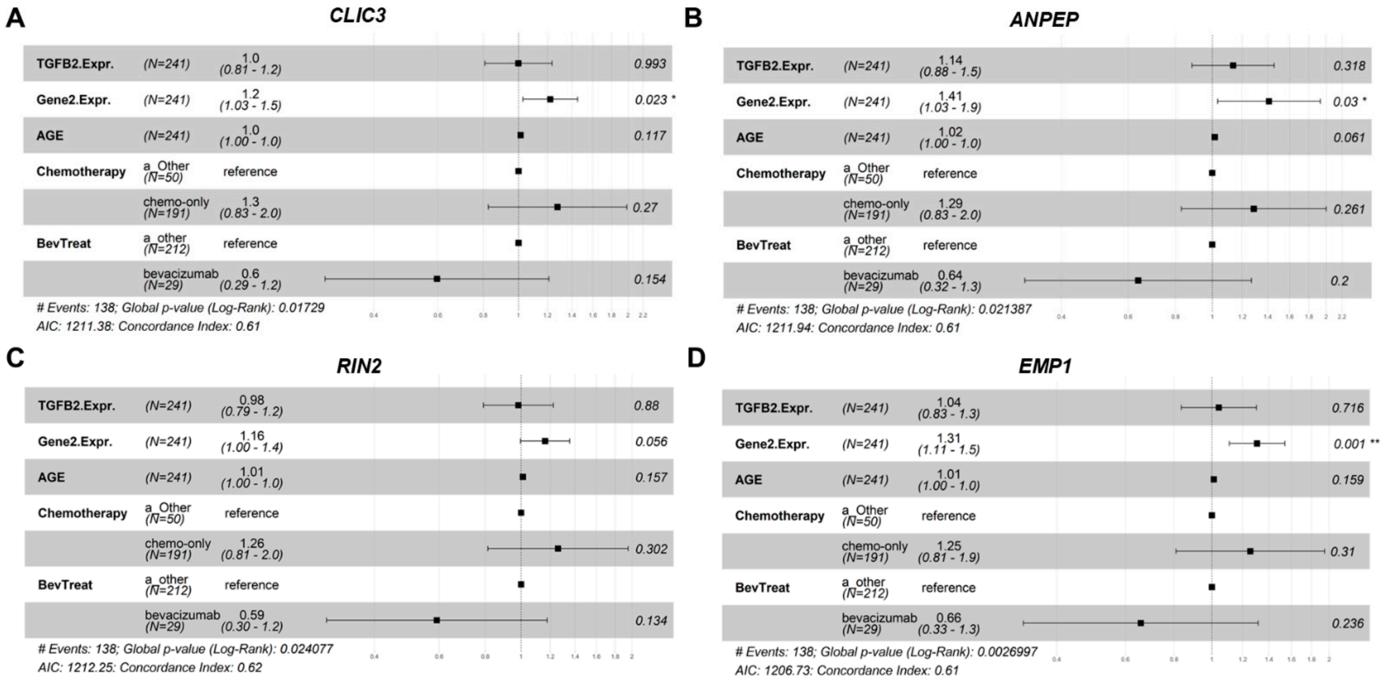

Figure S5. The impact of exposure to bevacizumab on *TGFB2* mRNA-independent prognostic markers identified from the multivariate Cox proportional hazards model in the TCGA dataset, cross-referenced with the KMplotter dataset. Multivariate analyses used the Cox proportional hazards model to assess the individual effects of *TGFB2*, Gene2 marker mRNA expression levels (A. *CLIC3*; B. *ANPEP*; C. *RIN2*; D. *EMP1*), and the *TGFB2*-dependent OS impact via the interaction term in the multivariate model (Model 3). This analysis was controlled for age at diagnosis (Mean age = 59 yr), treatment (chemo-only versus all other treatments) and exposure to bevacizumab. Model 3 included (i) *TGFB2* mRNA levels as a linear covariate expressed as Zscores (n = 241), (ii) The mRNA expression level for Gene2 mRNA levels as a linear covariate expressed as Zscores (n = 241), (iii) *TGFB2* by Gene2 interaction term to determine the dependency of Gene2 prognostic impact on *TGFB2* mRNA levels (n = 241), (iv). Treatment (Chemotherapy) comparing chemo-only (n = 191) versus other (n = 50) treatment regimens, and (v). Age expressed as a linear covariate (n = 241), and (vi). bevacizumab treatment (n = 29) versus others (n = 212). The forest plots depict the HR calculations (95% CI) for each of the parameters of the model (* indicates $p < 0.05$, ** indicated $p < 0.01$). Model fits were reported by the AIC and LRT p-values (“Global p-value (Log-Rank)”). [A] There was a significant increase in HR as *CLIC3* expression levels increased (HR (95% CI range) = 1.22 (1.03-1.45); p = 0.023). There was not a significant impact on HR for the *TGFB2* by *CLIC3* interaction parameter (HR (95% CI range) = 1 (0.76-1.3); p = 0.99). [B] There was a significant increase in HR as *ANPEP* expression levels increased (HR (95% CI range) = 1.41 (1.03-1.93); p = 0.03). There was no significant impact on HR for the *TGFB2* by *ANPEP* interaction parameter (HR (95% CI range) = 1.72 (0.92-3.19); p = 0.089). [C] There was a borderline significant increase in HR as *RIN2* expression levels increased (HR (95% CI range) = 1.16 (1-1.36); p = 0.056). There was not a significant impact on HR for the *TGFB2* by *RIN2* interaction parameter (HR (95% CI range) = 1.03 (0.85-1.24); p = 0.794). [D] There was a significant increase in HR as *EMP1* expression levels increased (HR (95% CI range) = 1.31 (1.11-1.54); p = 0.001). There was not a significant impact on HR for the *TGFB2* by *EMP1* interaction parameter (HR (95% CI range) = 1.22 (0.9-1.66); p = 0.194).

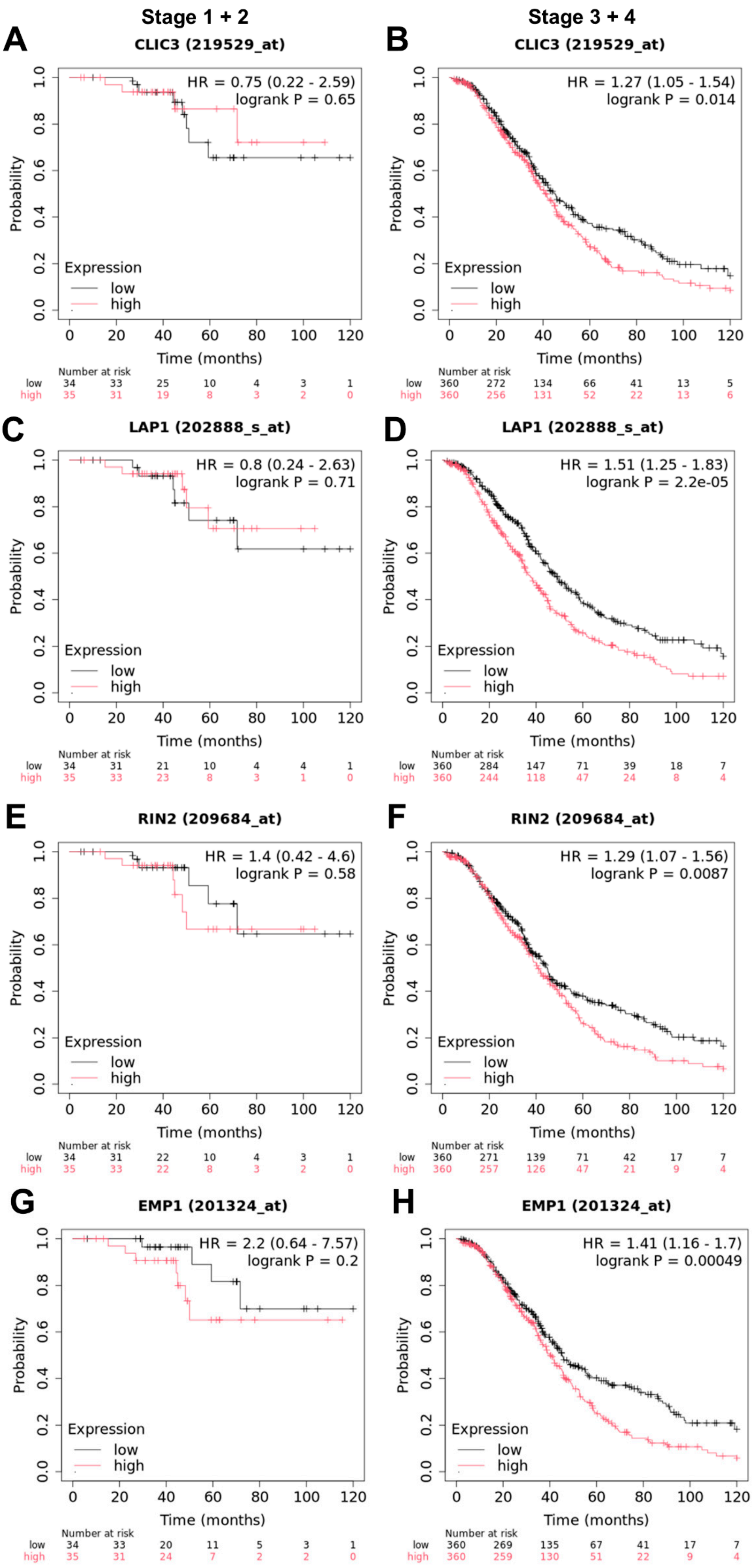

Stage 1 + 2
Stage 3 + 4
A
CLIC3 (219529_at)
HR = 0.75 (0.22 - 2.59)
logrank P = 0.65
B
CLIC3 (219529_at)
HR = 1.27 (1.05 - 1.54)
logrank P = 0.014
C
LAP1 (202888_s_at)
HR = 0.8 (0.24 - 2.63)
logrank P = 0.71
D
LAP1 (202888_s_at)
HR = 1.51 (1.25 - 1.83)
logrank P = 2.2e-05
E
RIN2 (209684_at)
HR = 1.4 (0.42 - 4.6)
logrank P = 0.58
F
RIN2 (209684_at)
HR = 1.29 (1.07 - 1.56)
logrank P = 0.0087
G
EMP1 (201324_at)
HR = 2.2 (0.64 - 7.57)
logrank P = 0.2
H
EMP1 (201324_at)
HR = 1.41 (1.16 - 1.7)
logrank P = 0.00049
Probability
Time (months)
Expression
low
high
Number at risk

Figure S6. Kaplan Meier comparisons Taxol-treated patients stratified according to cancer stage in an independent KMplotter dataset for *TGFB2*-independent biomarkers. We compared high versus low expression of prognostic marker genes (median cut-offs for A. *CLIC3*; B. *ANPEP*; C. *RIN2*; D. *EMP1*) for patients diagnosed with stage 1+2 (n = 69) and stage 3+4 cancers (n = 720).

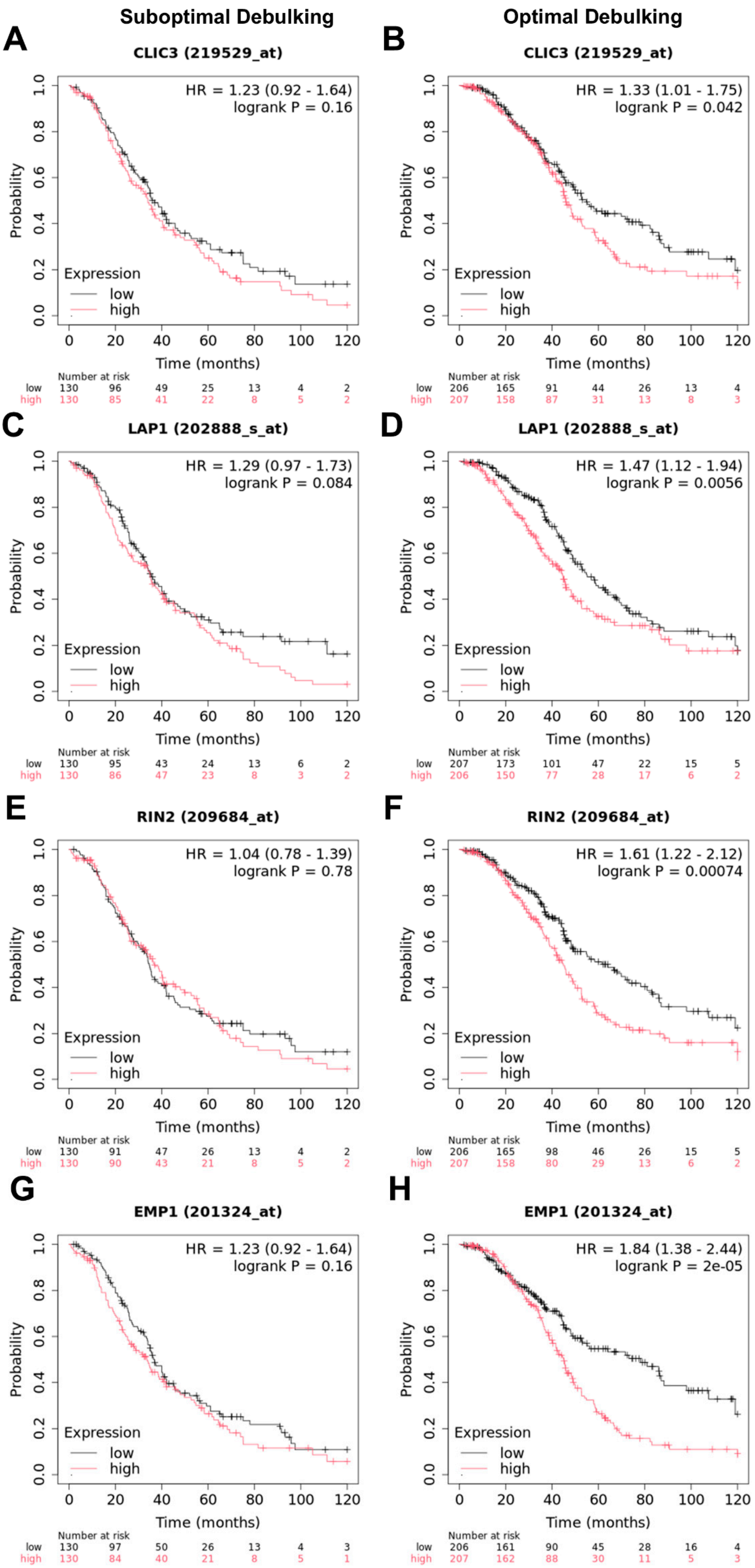
Suboptimal Debulking
Optimal Debulking
A
CLIC3 (219529_at)
HR = 1.23 (0.92 - 1.64)
logrank P = 0.16
Number at risk
low 130 96 49 25 13 4 2
high 130 85 41 22 8 5 2
B
CLIC3 (219529_at)
HR = 1.33 (1.01 - 1.75)
logrank P = 0.042
Number at risk
low 206 165 91 44 26 13 4
high 207 158 87 31 13 8 3
C
LAP1 (202888_s_at)
HR = 1.29 (0.97 - 1.73)
logrank P = 0.084
Number at risk
low 130 95 43 24 13 6 2
high 130 86 47 23 8 3 2
D
LAP1 (202888_s_at)
HR = 1.47 (1.12 - 1.94)
logrank P = 0.0056
Number at risk
low 207 173 101 47 22 15 5
high 206 150 77 28 17 6 2
E
RIN2 (209684_at)
HR = 1.04 (0.78 - 1.39)
logrank P = 0.78
Number at risk
low 130 91 47 26 13 4 2
high 130 90 43 21 8 5 2
F
RIN2 (209684_at)
HR = 1.61 (1.22 - 2.12)
logrank P = 0.00074
Number at risk
low 206 165 98 46 26 15 5
high 207 158 80 29 13 6 2
G
EMP1 (201324_at)
HR = 1.23 (0.92 - 1.64)
logrank P = 0.16
Number at risk
low 130 97 50 26 13 4 3
high 130 84 40 21 8 5 1
H
EMP1 (201324_at)
HR = 1.84 (1.38 - 2.44)
logrank P = 2e-05
Number at risk
low 206 161 90 45 28 16 4
high 207 162 88 30 11 5 3
Probability
Time (months)
Expression
low
high
0.0
0.2
0.4
0.6
0.8
1.0
0 20 40 60 80 100 120

Figure S7. Kaplan Meier comparisons Taxol-treated patients stratified according to debulking in an independent KMplotter dataset for TGFB2-independent biomarkers. We compared high versus low expression of prognostic marker genes (median cut offs for A. *CLIC3*; B. *ANPEP*; C. *RIN2*; D. *EMP1*) for patients with optimal debulking (*n* = 413) and patients with suboptimal debulking (*n* = 260).
